# The Ethics of Artificial Intelligence for Intelligence Analysis: a Review of the Key Challenges with Recommendations

**DOI:** 10.1007/s44206-023-00036-4

**Published:** 2023-04-05

**Authors:** Alexander Blanchard, Mariarosaria Taddeo

**Affiliations:** 1 The Alan Turing Institute, London, UK; 2 Oxford Internet Institute, University of Oxford, Oxford, UK

**Keywords:** Artificial intelligence, Intelligence analysis, National security, Digital ethics, Augmented intelligence

## Abstract

Intelligence agencies have identified artificial intelligence (AI) as a key technology for maintaining an edge over adversaries. As a result, efforts to develop, acquire, and employ AI capabilities for purposes of national security are growing. This article reviews the ethical challenges presented by the use of AI for augmented intelligence analysis. These challenges have been identified through a qualitative systematic review of the relevant literature. The article identifies five sets of ethical challenges relating to intrusion, explainability and accountability, bias, authoritarianism and political security, and collaboration and classification, and offers a series of recommendations targeted at intelligence agencies to address and mitigate these challenges.

## Introduction

National intelligence and law enforcement agencies ('intelligence agencies'), particularly those within mature digital societies, have begun to identify artificial intelligence (AI) as a key technology for maintaining advantage over adversaries and protecting against threats. The UK’s Government Communications Headquarters (GCHQ), for instance, has recently stated that “AI capabilities will be at the heart of our future ability to protect the UK” (GCHQ, 2021, 4). In the USA, the National Security Commission on Artificial Intelligence stated that “AI will revolutionize the practice of intelligence”, and that “there may be no national security function better suited for AI adoption than intelligence tradecraft and analysis” (NSCAI, 2021, 23). The Central Intelligence Agency (CIA) has stated that it is working on over “100 AI initiatives”, which it foresees continuing into the future (Vincent, 2019).

There is a number of potential and actual uses of AI across different agencies, including but not limited to the use of AI for the automation of administrative and organisational processes, the use of AI for cyber-security processes (including the management of analysts), and the use of AI for intelligence analysis, otherwise known as “AI-augmented intelligence” (see Babuta et al., 2020, vii). The adoption of AI for intelligence analysis enables intelligence agencies to meet the deluge of data created by digital communications and so using AI to facilitate the analysis of data will prove a key strategic advantage. As has been outlined:

“Future intelligence tradecraft will depend on accessing data, moulding the right enterprise architecture around data, developing AI-based capabilities to dramatically accelerate contextual understanding of data through human-machine and machine-machine teaming, and growing analytic expertise capable of swimming and navigating in enormous data lakes” (Weinbaum & Shanahan, 2018, 5–6).

This article focuses on the use of AI for augmented intelligence analysis, exploring its most common uses and the relevant ethical challenges, as identified through a qualitative systematic review (Grant & Booth, 2009).^1^ Section 2 outlines what augmented intelligence analysis is. Section 3 provides a review of the ethical challenges that have been reported as associated with the use of augmented intelligence analysis and offers a series of recommendations targeted at intelligence agencies to address and mitigate these challenges. Section 4 concludes the article.

There are three limitations in addressing the ethical challenges of augmented intelligence analysis. The first is that the uses of AI by intelligence agencies are mostly secretive. Research for this article has had to draw on publicly available information. While much can be inferred from such sources, particularly from defence contracts (see Techjournalist, 2020), this nevertheless limits the extent of reporting on existing use of AI by intelligence agencies.

The second limitation is generated by the novelty of the field. While the field of AI has a long history (Wooldridge, 2020), the applications that can be made of recent developments, such as machine learning and deep learning, are only just beginning to be understood (Tsamados et al., 2021). This is equally the case for the use of these technologies for national security purposes. While the ethical challenges associated with using AI for data collection are comparatively well explored, the ethical challenges of using AI for intelligence analysis are only just being addressed. The scope for exploring the literature addressing these challenges is therefore limited.

Finally, while this article refers to the intelligence agencies of several countries, the literature covered refers predominantly to activities by the USA intelligence community. There are practical reasons for this: there is a wider body of literature available on augmented intelligence analysis as employed by US intelligence agencies. In addition, having preponderant intelligence capabilities, where the US leads international partners often follow. Focusing on the USA thereby enables consideration of future potential ethical issues in the use of AI in other national intelligence agencies. This will be particularly relevant where issues of interoperability arise between intelligence agencies of partner nations.

## What Is Augmented Intelligence Analysis?

Augmented intelligence analysis has been variously defined; but broadly speaking, it is the use of AI to:

“…enhance human intelligence rather than operate independently of or outright replace it. It is designed to do so by improving human decision-making and, by extension, actions taken in response to improved decisions” (IEEE, 2019).

Augmented intelligence analysis has been made possible by new developments in AI technology, most especially the development of machine learning and deep learning.^2^ These technologies have a range of current and envisaged applications to intelligence analysis, including for purposes of defence (Alderton, 2017; Brewster, 2021; Cornille, 2021; Marcum et al., 2017; Office of the Secretary of Defense, 2017, 19; Pellerin, 2017; US Navy, 2019; [Taddeo et al., 2021]), counterterrorism (Campedelli et al., 2021; Doyle et al., 2014; McKendrick, 2019; Rassler, 2021), policing and countering crime (Dixon & Birks, 2021; Eggers et al., 2019; GCHQ, 2021; Ni et al., 2020; Serious Fraud Office, 2020; Vegt et al., 2022), human rights monitoring and humanitarian uses (Freeman, 2021; Marin & Kalaitzis, 2020; Pizzi et al., 2021; Ryan & Van Antwerp, 2019), and intelligence-gathering oversight (Vieth & Wetzling, 2019).

The areas of application of AI in support of human decision-making for intelligence analysis are described below. Before delving into these applications, let us consider the concept of intelligence analysis to clarify the potential application of AI. “Intelligence analysis” is still contested in the relevant literature (Ish et al., 2021), with different authors and institutions providing different definitions. For example, Johnston (2005, 37) defines intelligence analysis as:

“[...] a socio-cognitive process, occurring within a secret domain, by which a collection of methods is used to reduce a complex issue to a set of simpler issues.”

Palvin (as cited in Akhgar & Yates, 2013, 181) stresses that intelligence analysis provides

“[...] solutions capable of efficient and thorough exploitation of huge data volumes stemming from the omnipresent sensing, communication, and information processing systems.”

The Central Intelligence Agency defines intelligence analysis as

“[...] the application of individual and collective cognitive methods to weigh data and test hypotheses within a secret socio-cultural context.”^3^

The UK government describes intelligence analysis as a way to

“[add] value through the process of taking known information about situations and entities of strategic, operational, or tactical importance and characterising the known and the future actions in those situations.”^4^

**Fig. 1:**
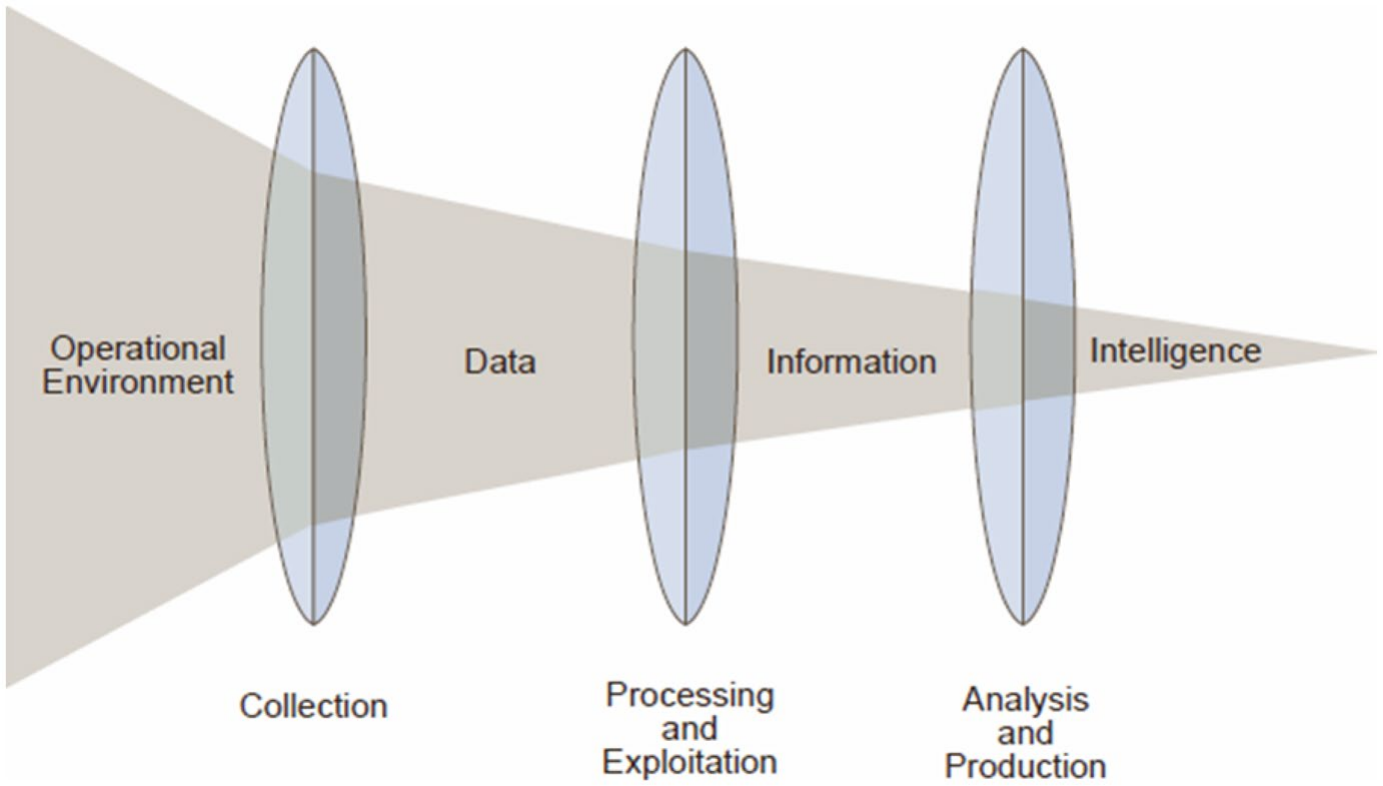
Intelligence analysis as a progressive refinement process (Defense Technical Information Center, DTIC; Department of Defense, 2013, I–2)

The rest of this article is agnostic with respect to a specific definition of intelligence analysis, but it agrees with the US Joint Intelligence report (Defense Technical Information Center, DTIC; Department of Defense, 2013) that intelligence analysis has the goal of refining data and information. In this context, data are conceived as raw unprocessed material; information is conceived as the well-formed combination of meaningful data (through processing and extraction, verification, and evaluation); intelligence is conceived as the combination and refinement of information to support decision-making (Floridi, 2012).^5^ This focus on the progressive refinement of data and information highlights the appeal of AI technologies to the IC. As Fig. 1 illustrates, the progressive refinement of data and information can be modelled as a cycle with a series of steps and processes.

**Fig. 2:**
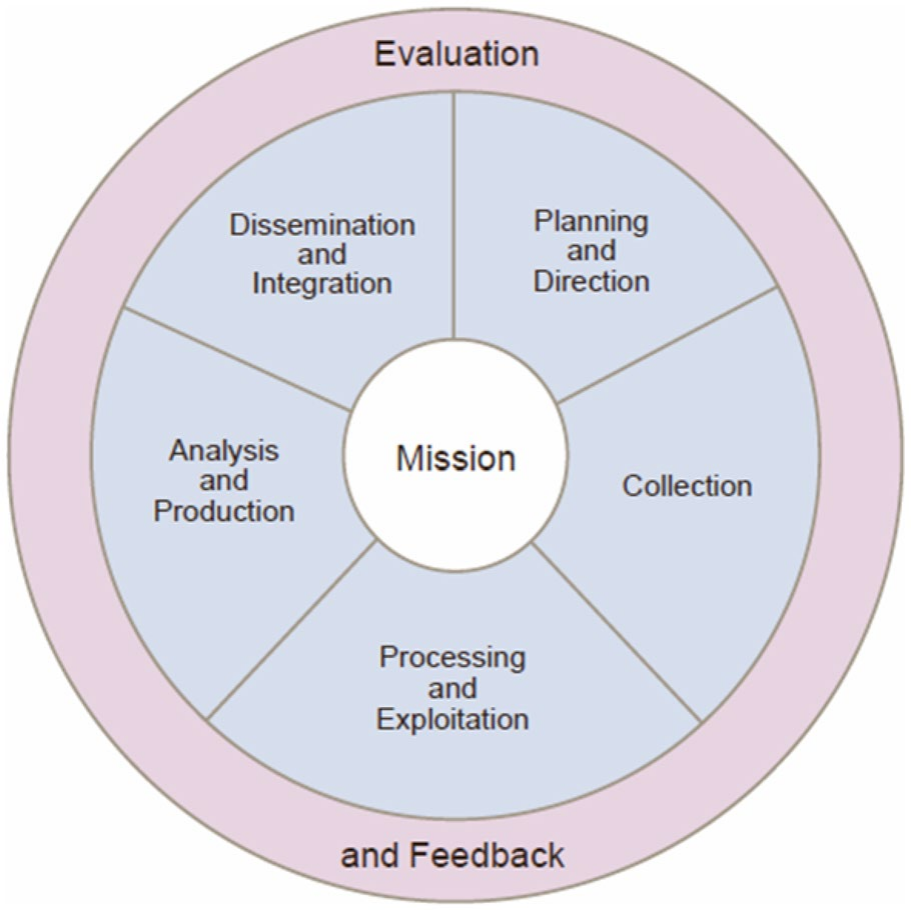
Intelligence cycle (Defense Technical Information Center, DTIC; Department of Defense, 2013, I–6)

Different intelligence agencies model this series differently. For example, the model proposed by the Office of the Director of National Intelligence (ODNI) indicates six steps: planning, collection, processing, analysis, dissemination, and evaluation. The model of the intelligence cycle provided by US Joint Intelligence report (DTIC; Department of Defense, 2013) also identifies six steps albeit differing slightly from those identified by ODNI (see Fig. 2).

The following summarises the steps and processes of the intelligence cycle as identified by different agencies:

1. Direction: whereby a decision-maker defines a set of priorities, usually as part of a threat assessment, which drive and shape the scope, approach, and goal of specific intelligence operations.
2. Collection: given the priorities defined at the direction step, an intelligence collection plan is defined, specifying collection methods, sources, and the need to gather data from other agencies.
3. Processing and exploitation: the process of extracting information from the collected data, including data labelling and curation.
4. Analysis: assessing the relevance of the processed data for the priorities identified at direction stage, and integration of these data with other data to extract relevant information and patterns.
5. Dissemination: depending on the level of threat, of the urgency, and of the type of information acquired, the finalised intelligence is labelled so to flag its priority with respect to other information and documentation.
6. Feedback: decision-makers share their feedback to update direction.

This article focuses specifically on the use of AI for processing and exploitation of data, and the analysis and production of information (Fig. 1). These are stages three to five of the summary above. Since each stage of the cycle influences those that both follow and precede it, the analysis of ethical implications at any stage must take a holistic approach. For instance, a clear understanding of the sources of data during collection (stage two) will determine the effective labelling and curation of those data (stage three), thereby determining its successful integration with other processed information (stage four). The focus on AI for intelligence analysis (stage four) is intended to address the comparative dearth of recommendations for this use of AI (see Verhelst et al., 2020). Stage five is included here as part of the analysis process, since the prioritisation and dissemination of information are constitutive of intelligence production.

**Fig. 3:**
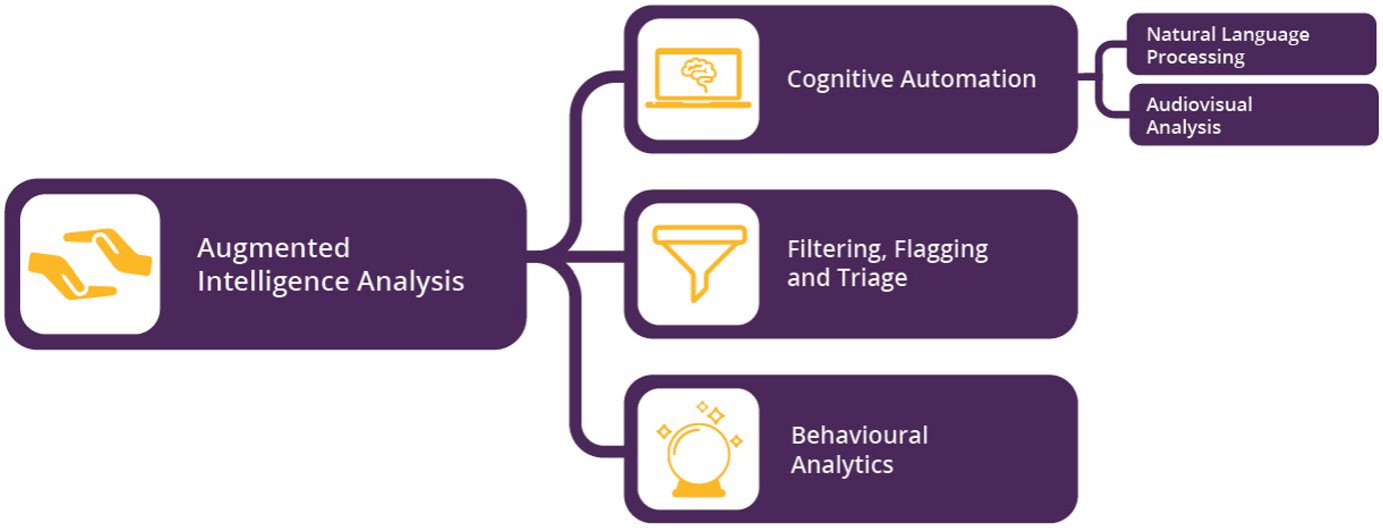
Areas of application of AI technologies to intelligence analysis (figure from Babuta et al., 2020, 8)

Existing literature suggests there are three key ways to use AI to support human analysts for intelligence analysis. Babuta et al. (2020) summarise these applications in Fig. 3.

**Cognitive automation** entails delegating to machines tasks which have been performed by humans thus far and which range across language processing, picking out patterns of speech, authorship attribution, classification and facial matching, and transcribing text from audio data for an analyst to search by using keywords or pre-set categories. For instance, a dominant trend in AI over recent years has been the development of ever-larger language models like the GPT-3 (OpenAI, 2021). Contemporary language models, underpinned by neural networks, can now provide sophisticated mimicry of reading comprehension, summarisation, and “common sense” reasoning (Open AI, 2019; Heaven, 2021; Rae et al., 2021).^6^ Language models can thereby aid the processing and exploitation of data by translating foreign language materials or by generating summaries of texts, thereby reducing the time required for the review of materials by intelligence analysts.^7^ Cognitive automation is therefore most likely to support the processing and exploitation of the data stage of the intelligence cycle. However, advances in cognitive automation may aid human analysts in the organisation of intelligence, too. For example, researchers at the UK’s Defence Science Technology Laboratory have developed a conversational agent to simplify information queries during criminal intelligence analysis. The use of an AI conversational agent can bypass a number of mundane tasks such as repeated information searches (Hepenstal et al., 2020).

**Filter, flagging, and triage** can be utilised for both the processing of data and the analysis of information. For instance, AI can be used for identifying connections among multiple sets of data in a way that is unfeasible for humans to do (GCHQ, 2021). Within this envisaged role, AI systems summarise sets of data, look for word matches, undertake sentiment analysis, and undertake object detection as part of the filtering process (Babuta et al., 2020).^8^ At the same time, AI can be used for filtering bulk information so that human operators are presented with the most analytically relevant information for making intelligence-based decisions. “Flagging” entails the AI system marking an item for the attention of, or review by, the human analyst. These uses of AI would likely function best when “deployed as a part of an interactive ‘human–machine team’ analysis workflow” (Babuta et al., 2020, 13; see also Ministry of Defence, 2018).

Using AI for this labour-intensive task relieves analysts from mundane work, freeing up time to devote to tasks requiring either specialised knowledge or the application of human-level intelligence. For example, deep neural networks have been used to analyse satellite imagery for surface-to-air missile sites across 35, 000 square-miles of southeastern China (Marcum et al., 2017). Typically, analysing satellite imagery for missile sites is a task undertaken by human analysts because hereto existing computer models could not identify these sites successfully. This created a capacity problem. As Director of the US National Geospatial-Intelligence Agency noted: “If we attempted to manually exploit all of the imagery we will collect over the next 20 years, we would need 8 million imagery analysts” (Alderton, 2017). The deep learning model developed at the Center for Geospatial Intelligence at the University of Missouri demonstrated the same statistical accuracy as humans (90%) while identifying missile sites eighty times faster than human analysts (Marcum et al., 2017).

**Behavioural analytics** relates to data processing and extraction. Prior to the development and massive adoption of AI technologies, the capacity of machines to identify data patterns was constrained by their programming. AI enables analysts to overcome these limits, as AI systems learn by interactions with the environment and other agents, extrapolating patterns from datasets through example rather than by following programmable rules. This makes AI particularly good at “digesting large amounts of data very quickly and identifying patterns or finding anomalies or outliers in that data” (Walch, 2020). Indeed, combined with other cognitive approaches, AI is capable of discovering “higher order connections” between data in a way not possible for humans.

AI has been used across a number of sectors for various tasks including: predicting rates of recidivism (Zhu, 2020), fraud detection by financial institutions and governments (West, 2021), and sales patterns and consumer preferences (Gal & Simonson, 2021). The Chinese government is reported to have developed a “geopolitical environment simulation and prediction platform”, which uses AI for big data analytics in order to provide Chinese diplomats with foreign policy suggestions (Prakash, 2019). In each case, AI models can be used to determine whether a given data point fits existing patterns or is an outlier or anomaly. The use of behavioural analytics by intelligence agencies would see them utilise these consumer-preference models to generate insights and predictions about certain events and individuals. This could then be used for

“[...] insider threat detection, predicting threats to individuals in public life, identifying potential intelligence sources who may be susceptible to persuasion, and predicting potential terrorist activity before it occurs” (Babuta et al., 2020, 13).

It is important to note that uses of terms like “prediction” need to be qualified. While commentators agree that AI can be used to forecast across various domains such as law enforcement (Evans, 2021; Raaijmakers, 2019; Rudin & Sloan, 2013), there is disagreement over whether AI can be used successfully to predict events like terrorist attacks. Used as part of human–machine teaming, behavioural analytics can help human analysts identify trends or characteristics indicating the probability of an individual participating in terrorism or being susceptible to radicalisation (Babuta et al., 2020, 14).^9^ But commentators are, on the whole, pessimistic that AI can be used successfully to predict events below population level, and there are no examples of AI models successfully predicting individual-level terrorist activities (Salganik et al., 2020; Roff, 2020b).

In this regard, a report by the House of Lords on the advent of new technologies in the justice system noted that vendors of predictive analytics systems are “over-claiming system capabilities for commercial advantage” (Justice and Home Affairs Committee, 2022, 69). Moreover, “even when accuracy rates advertised by providers are grounded in proper evaluations, […] they are not necessarily reflective of the technological solution’s actual performance once deployed” (Justice and Home Affairs Committee, 2022, 69).^10^ A significant part of the problem with using AI successfully for predictive analytics is the low quality of available data. Terroristic violence is comparatively infrequent, the corpus of historical data is small, and there is no consistent profile of a “terrorist”. As such, existing research has failed to “find valid nontrivial risk factors for terrorism” (Babuta et al., 2020, 15; Monahan, 2012). Moreover, prediction outputs remain problematic as they are based on inductive inferences (Bergadano, 1991). Insofar as these systems rely on inductive inferences, the value of their predictions needs to be considered carefully as, like any other inductive inference, they are limited by the problem of induction (Hume, 2009).^11^

## Ethical Challenges of Augmented Intelligence Analysis

The use of AI to augment intelligence analysis raises a number of ethical challenges that need addressing. The importance of ethics as a set of principles and guidelines alongside regulatory structures and oversights has been affirmed by the Director of GCHQ:

“…there are ethical rules and boundaries, and these should always be followed and upheld…our analysts are constantly reminded that it is not enough to be able to do something…it is not enough for it to be legal to do something…it must also be right to do something…” (Fleming, 2019)

Doing what is right, or indeed knowing what it is right to do, can be difficult in circumstances where there is a lack of accepted norms around the use of emerging technologies. For intelligence agencies, this may mean that novel capabilities introduced by these technologies alter the delicate balance that ought to be struck between protecting citizens and fostering their rights. Here, we consider a number of potential ethical challenges as found in current literature and make recommendations for addressing them. It is important to highlight that there is a dearth of literature considering the ethical challenges of employing augmented intelligence analysis. This is because the use of AI for augmented intelligence analysis is a novel phenomenon, and scholarly literature on the subject is currently outpaced by the emergence of these technologies.

In lieu of such work, the article draws on literature from the ethics of data collection and the ethics of using AI for predictive policing as they provide a useful touchstone for the ethics of augmented intelligence analysis. It is important to remain mindful of the limitations of applying this literature to understand the ethical implications of augmented intelligence analysis, as the ethical considerations applicable to (predictive) policing may not cover comprehensively other uses such as defence intelligence (Taddeo et al., 2021). That said, literature on predictive policing remains relevant to augmented intelligence analysis in so far as the latter often exacerbates existing ethical issues associated with the former.

This article was designed to be a qualitative systematic review of existing literature. As described in Grant and Booth (2009), this method for literature reviews is

“a method for integrating or comparing the findings from qualitative studies. […] It ‘looks’ for ‘themes’ or ‘constructs’ that lie in or across individual qualitative studies. The goal is not aggregative in the sense of ‘adding studies together’, as with a meta-analysis. On the contrary, it is interpretative in broadening understanding of a particular phenomenon” (p. 99, citing: Booth, 2006).

The data collection was conducted by querying Google Scholar and Scopus. To ensure the review of ethical challenges is wide-ranging, a number of phrases were used to query the two scholarly databases: “the ethics of artificial intelligence for augmented intelligence analysis”, “the ethics of artificial intelligence for intelligence analysis”, “the ethics of artificial intelligence for data collection”, and “the ethics of artificial intelligence for national security”. Results from literature searches were then selected manually to identify articles that could be placed at the intersection of the three categories; 153 articles were identified. After cleaning for duplicates, 131 texts were reviewed. Key themes are detailed below using 87 articles from the literature set. The selected literature was supplemented by material from existing author repositories to contextualise findings, and this included key texts in the field of intelligence studies, digital ethics, and artificial intelligence ethics. In addition, material was supplemented by a review of recent policy documents and research papers published or commissioned by organisations undertaking intelligence analysis. This pertained predominantly to governmental intelligence organisations constrained, for purposes of scope and foreign-language limitations, to intelligence organisations within the anglophone “Five Eyes” intelligence-sharing partnership: Australia, Canada, New Zealand, UK, and USA. Published texts from a small number of non-governmental organisations undertaking intelligence-based work were also included.

Lastly, while recommendations draw on literature from US and UK contexts, the recommendations are not made to address any specific organisation, institution, or public oversight body. This is so that the recommendations made here remain applicable across different national contexts.

### Intrusion

Within liberal democracies, intelligence organisations are tasked with protecting national security while respecting the rights and values commensurate with liberal democratic government. Such values include the right to a private life for every individual. A central issue in the ethics of intelligence operations, particularly data collection and analysis, is the acceptable level of intrusion against those rights and values. The advent of digital communications and the collection of bulk datasets have brought to the fore the question of permissible intrusion in new ways. As observed by the United Nations High Commissioner for Human Rights (2014, 3):

“[…] examples of overt and covert digital surveillance in jurisdictions around the world have proliferated, with governmental mass surveillance emerging as a dangerous habit rather than an exceptional measure.”

A central feature of the debate on the ethics of augmented intelligence analysis is whether it will mean greater or lesser intrusion of the data-subject and, therefore, whether it represents the potential for greater of lesser protection of privacy rights. One argument that is made is that augmented intelligence analysis has the potential to reduce levels of intrusion into private data because it reduces the quantity of data that needs to be “seen” by the data analyst (Babuta et al., 2020).^12^ In this regard, Omand and Phythian (2018, 24–25) argue that the level of intrusion is a technical question depending on the efficiency of algorithm used to filter data:

“Whether such techniques are compatible with privacy rights depends on how discriminating and efficient both the algorithms used to filter and discard unwanted material unseen (including the communications of those not the subject of the operation) and the selectors that pull out communications of intelligence interest from what remains.”

However, whether AI can diminish intrusion also depends on what counts as “intrusion” and at what point it begins. These questions were widely discussed after the Snowden revelations about bulk data collections by intelligence agencies, such as the NSA and GCHQ. Bernal (2016), for instance, argues that intrusion is not defined solely by the exposure of data to the human analyst but by their collection, storage, and processing (see also Kniep, 2019). This position concurs with UK’s 2011–2017 Independent Reviewer of Terrorism Legislation, who argued that in law^13^ there is “interference” with material not only when it is “read, analysed, and shared with other authorities but also when it is collected, stored, and filtered even without human intervention” (Anderson, 2016, 76). This legal position establishes that the use of AI in place of a human analyst would not necessarily diminish intrusion. An alternative view suggests grading the levels of intrusion, such as the 2015 Independent Surveillance Review distinguishing the relative impacts of the processes of data collection, retention, and analysis on privacy. The panel of the Independent Surveillance Review suggested that the issue of privacy needs “to be considered afresh at each stage” of activity entailing the use of data (Independent Surveillance Review, 2015, 108). Omand and Phythian seek to reconcile intrusion and harm by distinguishing “potential” from “actual” intrusion. Potential intrusion exists once data have been collected, and actual intrusion has taken place once those data have been analysed and exploited for information. They explain that:

“If innocent people are unaware that their communications have been intercepted, stored, and filtered out by computer, thus not ever seen by a human analyst, then the intrusion is potential, not actual, and the potential for harm to the individual negligible” (Omand & Phythian, 2018, 24–25).

We disagree that the potential for harm in such a scenario is “negligible.” First, while Omand and Phythian refer to “harm to the individual,” it is worth bearing in mind that there are collective harms done to marginalised groups rather than individuals per se (Mantelero, 2017; Tisne, 2021). Likewise, there may also be harms that are done to the social and political institutions that uphold substantive and procedural justice. Second, the harm that potentially results from the collection of large datasets does not depend on the data subject’s knowledge of those practices. Contrary to the principle of data minimisation, the use of AI for intelligence analysis also has the potential to create “data creep,” whereby as the capacity for processing data increases, for instance through the use of machine learning, so will practices of data collection (United Nations High Commissioner for Human Rights, 2021). The fact that quantities of data that would not have been collected had the AI not been employed as part of intelligence analysis is where the dangers for increased levels of intrusion lie. In the case of data creep, it is the properties of AI itself that will drive the collection of ever-greater quantities of data, of data types, and data sources. This is because AI requires a large amount of data as inputs to operate effectively. As GCHQ (2021, 12) has noted: “AI does not work well when tackling ambiguous, broad challenges particularly if there is inadequate data on which it can train and learn.” Weinbaum and Shanahan (2018, 6) describe an “ironic dilemma” of the digital age whereby “there is too much data for humans to search effectively for needles, yet not enough accessible data from which to draw and validate useful intelligence.” This could lead to a situation where intelligence organisations already collecting large amounts of data find they do not collect enough to generate valid insights or useful information and are thereby moved to expand their collection programs.

Likewise, Kniep (2019) argues that if the automated collection and storage of data that is already undertaken by intelligence agencies constitutes intrusion, then the algorithmic analysis of data must deepen that intrusion even further. However, if using AI to analyse collected data did entail intrusion, that need not be a problem *per se*. The important question is whether that intrusion is justified, necessary, and proportionate. In this regard the UK Supreme Court has set out a test to determine whether an infringement of a fundamental right (e.g. an invasion of privacy and data protection) is acceptable. This includes that the objective be important enough to justify an infringement of human rights, that less intrusive means do not exist to fulfil the objective, that the intrusion is “rationally connected” to the objective, and that a “fair balance [is] struck between the rights of the individual and interests of the community” (Babuta et al., 2020, 23). McKendrick (2019) has argued that, on these terms, the use of AI for tasks such as predictive analytics would be impermissible. First, she argues that the use of AI would be “inherently disproportionate” because the vast majority of data required to generate valid trends for predictive analytics would be “generated by people who are not of interest to intelligence services.” The use of AI for predictive analytics would constitute a “surveillance measure applied to the whole population” (McKendrick, 2019, 14–15). Second, AI for predictive analytics fails to meet the necessity clause. The failure to meet this clause need not be because blanket retention is wrong in principle, but because blanket retention “cannot be linked to a specific legitimate objective with a clear causal relationship to the policy” (McKendrick, 2019, 15–16).

The question is then which data are collected, accessed, and analysed given a specific policy purpose? The need to have clear criteria as to what data are collected, who accesses them, and how these data are collected and stored became clear during the Covid-19 pandemic when track-and-trace apps started to be developed and used to monitor and limit the spreading of the virus (Morley et al., 2020). As such, the following recommendations are suggested to limit the intrusion on individual and group privacy and hence the erosion of it that the use of AI for intelligence analysis may pose.

**Purpose-oriented data collection and analysis**. In order to meet the principles of necessity and proportionality, data used to extract intelligence-relevant information should only be collected and analysed on the basis of an assessment concerning the more relevant type of data for a given purpose. The assessment should be based on the likelihood of a specific type of data revealing relevant information for a given purpose and should be context-dependent. For instance, the use of AI for undirected surveillance for defence purposes will be unacceptable in the context of domestic policing. The assessment should therefore also include comparisons among different types of data and choose those data which would lead to similar outcomes in terms of relevancy and accuracy of the extracted information but lead to lighter erosion of individual privacy. If implemented, this recommendation would improve step 2 of the intelligence cycle described in Section 2, for it asks intelligence agencies to specify criteria to assess the relevancy of a given data set for a given purpose, on top of clarifying their methods and sources. At the moment, the relevancy of the data is mentioned in step 4 as part of the analysis. With this recommendation, this article suggests that relevancy of data for a given purposes needs to be assessed much earlier in the process. More importantly, this assessment should be conducted before, not after, collection. This would make breaches of the principle of proportionality less likely, as only relevant data would be collected and would also avoid “data creep”, as data would be collected for its value in fulfilling the obligations of intelligence agencies and not for the effective functioning of AI.^14^

### Explainability and Accountability

The principle of explainability is central to the ethics of AI (Coeckelbergh, 2020). Broadly, for a given audience, an “explainable” AI is “one that produces details or reasons to make its functioning clear or easy to understand” (Baber et al., 2021, 10). Explainable AI thereby allows decision-makers to provide a rationale for a given decision. A report for the House of Lords affirms the importance of the principle of explainability for democratic processes, stating that:

“The development of intelligible AI systems is a fundamental necessity if AI is to become an integral and trusted tool in our society […] We believe it is not acceptable to deploy any artificial intelligence system which could have a substantial impact on an individual’s life, unless it can generate a full and satisfactory explanation for the decisions it will take” (Select Committee on Artificial Intelligence, 2018, 40).

The emphasis on explainability is for its importance for the citizen in holding decision-makers to account. This is no less the case for intelligence agencies where intelligence analysis can be used to inform the rationale for decisions with potentially severe consequences, and so must be justified and explained.

The challenge for explainability has become more pressing as increasingly more complex AI systems are designed and used. In rule-based AI systems such as those employing decision-trees, humans can, in principle, explain the decision process that leads to certain outputs on the basis of its programming. On this basis, humans can give an account of and take responsibility for the outputs produced by such models (Coeckelbergh, 2020, 116). Newer AI technologies envisaged in the use of augmented intelligence analysis, such as neural networks and machine leaning, are often black box systems, i.e. the decision process through which these systems elaborate their outputs is obscure to humans, as in the case of neural networks (Bathaee, 2017).

Vogel and colleagues (2021) have described concerns about how lack of explainability impacts accountability within intelligence agencies and have highlighted that the question of explainability has as much to do with the competencies and knowledge possessed by the analyst as with the transparency of the system. AI models have “idiosyncrasies” and “blind spots” in their processing of data, leading to incomplete or even misleading information for the intelligence analyst. While programmers may be able to scrutinise these idiosyncrasies, to the intelligence analyst, this may remain opaque (Vogel et al., 2021). A report by Deloitte commissioned by the US government also stated that intelligence agencies must create trust between analysts and tools for augmented analysis. Such trust will allow analysts to “stand behind their assessments even when powerful people may disagree…” (Mitchell et al., 2019, 9). Analysts are likely to be hesitant to defend outputs from a system they cannot trust (see also Parasuraman & Riley, 1997; Taddeo, 2017). Vogel and colleagues (2021, 840) suggest that to maximise explainability of AI outputs, intelligence analysts using AI must be equipped with three separate capabilities: the first entails the capacity to “productively leverage […] algorithmically produced assessments.” Second the capability to recognise limitations in both the data used by AI technologies and limitations in how those technologies handle the data. Third is the capacity to identify and leverage alternative sources of data to compensate for blind spots in AI technology. Such recommendations align with other contributions which call for the analyst to remain “in the loop.” Mitchell and colleagues (2019) have argued for a number of measures for keeping analysts “in the loop,” including interfaces that provide representations of how AI models arrive at their conclusion(s), as well as simulated AI outcomes which enable analysts to scan the data underpinning those outcomes. Such measures “would allow for much more reliable, trusted data and would yield more reliable analysis being presented to war fighters and decision-makers.” The trust generated by these measures, they argue, will ease the incorporation of the AI system into analysts’ workflow (2019, 9).

However, there is a possibility that this requirement for testing, evaluative, and auditing procedures will likely stand in tension with the time and labour reductions promised by augmented intelligence analysis. While this is true in principle, the friction between transparency requirements and shortage of human resources is less evident in practice. This is because measures to mitigate the consequences of lack of transparency need not involve analysts directly. They can be, and in some cases should be, outsourced to third parties. Lack of transparency is characteristic of deep learning models. While there are technical ways to reduce it, the most effective solutions come from overseeing the use of AI technologies (Floridi et al., 2022). This article offers two recommendations to mitigate the risk related to black box AI.

**Use interpretable AI**. This recommendation focuses on the type of AI models that should be privileged for augmented intelligence. Often, the debate on the lack of transparency hinges on a dichotomy, namely, accuracy vs transparency of AI (Tsamados et al., 2021). According to this view, less explainable models are more accurate and thus it can be necessary to sacrifice transparency (and with it accountability) to ensure more accurate results, especially when key aspects like health or security are at stake. This article follows Rudin (2019, 207), agreeing that;

“[…] this [dichotomy] is often not true, particularly when the data are structured, with a good representation in terms of naturally meaningful features. When considering problems that have structured data with meaningful features, there is often no significant difference in performance between more complex classifiers (deep neural networks, boosted decision trees, random forests) and much simpler classifiers (logistic regression, decision lists) after preprocessing.”

Because of this, the first recommendation to limit the ethical risks posed by the lack of transparency is to resort to interpretable AI models. This is because interpretable models can provide explanations “faithful to what the model actually computes” (2019, 206). This recommendation refers to step 3 of the intelligence cycle described in Section 2, as it offers a pragmatic way to improve the transparency of the tools used for data exploitation. The second recommendation focuses on deployment practices of AI and thus addresses the entire lifecycle of AI as used by intelligence agencies.

**Ethics-based auditing**. The learning capacity of AI implies that it may develop new, unforeseen behaviour from its interactions with the environment. These could be perfectly correct behaviour, i.e. the new behaviour is a coherent outcome of the functioning of the machine, and it could also be the result of an error in the system or of a third-party manipulation (Taddeo et al., 2019). In all cases where unwanted consequences can be foreseen, the mitigation is to identify these behaviours as soon as possible to intervene, stop, and correct them. As not all possible outcomes of AI systems are predictable (Holland Michel, 2020; Taddeo et al., 2022), it is also important to monitor the deployment of AI systems to assess points of failure and correct these before future deployments. To this end, it is crucial that the AI for augmented intelligence is audited to identify unethical behaviour in a timely and effective manner. The ethics-based auditing should concern the AI system, the decision processes in which it is embedded, and the organisation which uses this technology (Mökander & Floridi, 2021).

The first step to establishing ethics-based auditing for augmented intelligence analysis will require the intelligence agency to identify and state the ethical principles that shape their conduct. These should be clear, low-granularity principles that can offer specific guidance to analysts and whose violation is clearly identifiable. Such a principle could be, for example, maintaining human autonomy in Human-Machine Teaming (HMT) by ensuring an appropriate level of training for the human agent and opportunity for this agent to question and consider alternatives to the outcomes posed by the AI system. One may also imagine similar principles concerning the protection of individual rights, transparency, or accountability. Once these principles have been identified and shared (at least internally), they become the benchmark to assess whether and to what extent a given deployment of AI respects them and, if not, at which stage of the life cycle the breach occurs and for what reasons, e.g. inadequate training, lack of transparency of a specific model, or too generic criteria for the assessment of data relevancy leading to breaching the proportionality principle. To do so, an auditing procedure needs to be specified. To this end, this article refers to the auditing protocol proposed in Floridi et al. (2022).

The protocol proposed in Floridi et al. (2022) rests on a process view of AI systems and assesses their entire life cycle, i.e. design, development, evaluation, operation, and retirement to check adherence to the principles and values, as defined by the organisation using the AI system.^15^ This protocol identifies four stakeholders: top management responsible for AI, product owner, project manager, and data scientist. It has six stages:

“[...] at each stage, the requirements consist of two aspects: (1) organisational governance and (2) the use case for the AI system in question. Each requirement is linked to an actor who is best placed to ensure and confirm that the requirement in question is met. For many requirements, supporting evidence will be requested. Overall, there are 40 items to complete in the protocol” (Floridi et al., 2022, 18).

If used in an intelligence agency, this protocol would allow for clarity of accountability, and, at the very least, map those who are held accountable for meeting specific requirements. It would also favour an assessment of the HMT using AI systems and, ultimately, of the entire organisation, rather than focusing only on the technology. For each step of the intelligence cycle, it would facilitate the identification of problems and mistakes and offer an opportunity to address them before the next iteration.

### Bias

The problem of bias in AI systems is well established. All AI models demonstrate inherent biases regardless of the steps taken to remove bias from data chosen to train the model. Recognising and monitoring for bias, as well as having a plan to mitigate bias, are important because otherwise it can lead to outcomes that perpetuate harmful societal biases (Cath et al., 2018). We focus on two aspects: bias in society and bias in hybrid teams. When considering augmented intelligence, bias is problematic as it may lead to wrong conclusions and, thus, to the unjustified breaching of individual rights or perpetuate the harmful biases that exist in wider society. It may even be the case that bias deepens societal injustice as the outputs of algorithms are mistakenly taken to be neutral rather than the product of “subjective decisions” around data inputs, algorithmic parameters, set by the “machine learning practitioner” (Cummings & Li, 2019). In each case, political and societal justice can be harmed.

Roff (2020b), for instance, undertook an analysis of the components that comprised the early model-based event recognition using surrogates (EMBERS) predictive analytics. The system functions by ingesting a number of open-source data streams (such as social media content and local news outlets) and uses AI to generate real-time predictions about population-level events such as civil unrest, election outcomes, and disease outbreaks. Funded by the US Intelligence Advanced Research Projects Activity, EMBERS was earmarked as a potential precursor system for predicting terrorist attacks (Doyle et al., 2014, 185).

A subcomponent of EMBERS attributes sentiment scores to text fed to the system. To do this, EMBERS relies on a dataset called the “affective norms for English words”, otherwise known as “ANEW.”. ANEW was developed in the 1990s to provide a “metric” of emotional affect to a given set of words. However, as Roff (2020b) describes, researchers compiling ANEW developed this metric by asking college students to provide their emotional response to sets of words using “emojis” representing a range of nine emotions. The cumulative score for each word was taken as the “sentiment” represented by the given word (Roff, 2020b; see also Bradley & Lang, 1999).

This generates limitations in using ANEW for sentiment analysis of words. First, the sentiment analysis was conducted in an English lexicon. EMBERS, however, has been used to assess sentiment in Latin American countries. While it is possible to translate the words, it does not mean that the translated word will carry the same sentiment as in the English lexicon. Second, the sentiment data, being collected from college students in the US, represented a very specific sample not necessarily generalisable to other contexts. Words like “graduate” and “diploma”, for instance, had some of the highest scores in the dataset. Third, the dataset contained deeply harmful biases, particularly relating to gender norms and stereotypes. There were a greater number of words related to women than to men, and those related to the former were predominantly pejorative while those relating to the latter had predominantly positive connotations. This imbalance is troubling “from an instrument design perspective.” More troubling was that the affective division of male and female by the word scoring demonstrated a “valuation of heteronormative roles” and “underlying connotations of devaluing stereotypes” (Roff, 2020b, 4).

A key failing of the developers of the EMBERS system is that they did not consider whether the ANEW lexicon “was appropriate for their purposes” (Roff, 2020b). It is crucial to explore these limitations, particularly around biases, because of the potentially severe ramifications on social justice, for example, if predictive systems are used to inform foreign policy (Roff, 2020a, 6). Bias is also problematic insofar as, if not dealt with properly, it can undermine the use of AI by human analysts. This can be a consequence of naïve deployment, where analysts are not fully aware of possible biases of AI but are asked to trust these systems and to take accountability for their behaviour. This requires analysts to be sensitised to the biases that inhere to AI models and which are introduced into outputs through the AI system. Mechanisms for control and evaluation of these systems will have to mitigate and correct for bias as far as possible (Vogel et al., 2021). Regarding this, Vogel and colleagues make two recommendations. First is that intelligence agencies take steps to monitor the way that “algorithms are constructed, the kinds of training data that are used, and the various technical constraints that can be introduced through this entire process” (Vogel et al., 2021, 836). Second, they recommend that analysts using augmented intelligence systems should be given training and tools to enable greater awareness about the biases existing in algorithms and to recognise the limits of these technologies with respect to these biases. This would include mechanisms for questioning the outputs of algorithmic analysis, mechanisms for redress where analysts are unfairly held accountable for algorithmic bias, explanation for the procedures followed by the algorithm, descriptions of the data-gathering process, and the adoption of rigorous methods to validate methods and results.

In addition to these two recommendations, risks related to bias in society, particularly to social justice, must be considered and mitigated when using AI for augmented intelligence. To this end, the following recommendation is offered:

**Check your data**. Analysts relying on AI should be able to access the relevant data set and have adequate technical competences to assess whether protected characteristics are present and how they are 'read' by the AI system. AI systems should also run on synthetic data to ensure that risks of training a system on biased data are reduced to a minimum. In addition, teams that are tasked with checking the data should be made up of a diverse demographic to facilitate the identification of risks arising from bias and their impact on minority groups.

This recommendation addresses step 4 of the intelligence cycle described in Section 2, as it introduces the need to focus on bias in the analysis of datasets.

### Authoritarianism and Political Security

In liberal democratic systems of government, there is an expectation that the use of AI technologies by intelligence agencies will conform to existing oversight as well as wider principles for the ethical use of AI. This may not be the case when considering uses of augmented intelligence by authoritarian regimes. For instance, the Chinese government has been reported as embracing facial recognition and video behavioural analysis for identifying wanted criminals at public events and for identifying ethnic minority groups (Roberts et al., 2020). Huawei has filed patents for using facial recognition technology to identify Uighur minorities in public spaces (Harwell & Dou, 2020). The patent details the use of deep learning models to identify the features of individuals filmed or photographed in the street. The development of this technology meets a technical requirement for working with the Chinese Ministry of Public Security that video surveillance be capable of detecting ethnicity (Kelion, 2021). Brundage and colleagues (2018) warned in their report that the use of augmented intelligence in this way could have severe repercussions for what they call “political security”. Political security is likely to be impacted as authoritarian regimes “take advantage of improved capacity to analyse human behaviours, moods, and beliefs on the basis of available data” (Brundage et al., 2018, 6).

Moreover, for states that lack the breadth of infrastructure or resources of the Chinese government, the advantage of AI is for its capacity to 'upscale' intelligence analysis without the cost of recruiting additional analysts or developing a larger, more costly, intelligence architecture. As Brundage and colleagues note, hereto existing surveillance system may easily gather data on citizens, but extracting information from those data and turning that information into intelligence can be too costly for many authoritarian regimes (Brundage et al., 2018, 47). Once fully integrated with existing mechanisms of control, AI systems may

“[…] improve the ability to prioritise attention (for example by using network analysis to identity current of potential leaders of subversive groups) and also reduce the cost of monitoring individuals (for example using systems that identify salient video clips and bring them to the attention of human agents)” (Brundage et al., 2018, 47).

Indeed, these concerns are most pertinent to authoritarian regimes, but there is a need to remain aware that these technologies may also undermine the ability of liberal democracies to sustain political freedoms. The availability today of structured and unstructured data is so extensive as to “overwhelm all previous forms of analytic tradecraft and pattern recognition” (Weinbaum & Shanahan, 2018, 4). This transformation results from both the growing demand for information about individuals (such as terrorists, international criminals) rather than states *per se* post 9/11, and the growth of digital communications able to supply data about those individuals in ways not previously thought possible (Omand & Phythian, 2018, 142). This rise in the supply of, and demand for, digital private communications has been accompanied by the increasing availability of open-source data for intelligence analysis (Janjeva et al., 2022). While AI will prove pivotal for extracting information from this glut of data, the power it offers for improved sense-making has the potential to transform the relationship between state and citizen in ways not yet fully understood. This, as McKendrick (2019, 14) has argued, may require advising measures for safeguarding goods and freedoms not normally associated with data collection (such as privacy) but are nevertheless “critical to democratic functioning, such as those of expression and association.”

In this regard, this article stresses that the way in which the problem of explainability addressed above converges with that of political security. As indicated by the House of Lords report (Select Committee on Artificial Intelligence, 2018, 39), if it is not possible in principle for institutions to explain to a wider public how AI is functioning in the decision-making process, can the public be said to be consenting to what those institutions are doing? This has ramifications in countries like the UK where policing is said to exist by public consent and where the existence of police powers is meant to be dependent on the public approval of those powers (Home Office, 2012). It also has ramifications for democratic deliberation not just about the outcomes of decision-making processes but the very legitimacy of decision-making processes themselves, in turn undermining faith in democratic procedures and institutions.

We propose that democratic institutions take on the essential role for setting and maintaining limits in the use of augmented intelligence analysis, practicing vigilance, so that a clear demarcation between democratic and authoritarian uses of these systems persists. A great example in this sense comes from the draft of the EU AI Act,^16^ which forbids uses of AI for facial recognition and focuses strongly on the risks that the use of AI poses to individual rights. The following recommendations take this approach:

**Justified uses of AI**. As Floridi and colleagues (Floridi et al., 2020, 1773) stress:

“[...] it is important to acknowledge at the outset that there are myriad circumstances in which AI will not be the most effective way to address a particular social problem. This could be due to the existence of alternative approaches that are more efficacious or because of the unacceptable risks that the deployment of AI would introduce.”

Hence, it is crucial that the (non) adoption of AI is justified to ensure that AI solutions are not being underused, thus creating opportunity costs, or overused and misused, thus creating risks. Similarly, the decision to (or not to) resort to AI should be overridable should it become clear that it leads to excessive breaching of rights or the securitisation of right (Ad’ha Aljunied, 2019). A third independent body should be tasked with assessing the cost/benefit analysis underpinning the justification of AI use. While the assessments remain confidential, this body should be publicly identifiable and share accountability with intelligence agencies for misuses and overuses of AI for intelligence analysis. Given the question of consent outlined above, this body should also be able to explain to a wider public how AI systems are used by ntelligence agencies. Given the nature of intelligence agencies and their mandated level of secrecy, it is neither possible nor necessarily desirable that all processes using AI are made fully public. But it will require that this body can explain the potential ramifications of using a given system on democratic rights and civil liberties. This will also require a consultation process with the relevant organisations about which internal processes relating to the use of AI can and cannot be made more transparent. This recommendation addresses step 1 of the intelligence cycle described in Section 2. It adds an extra dimension to the decision-making process, whereby the use of AI is not a default decision but the outcome of an assessment, considering the advantages but also the ethical risks that the use of this technology poses.

### Collaboration and Classification

A number of AI-enabled platforms are being developed so as to facilitate better interoperability among intelligence analysts by increasing the processing and transmission of information. In the US, the development and implementation of platforms for greater collaboration has been a longstanding aim following the intelligence failures highlighted by the 9/11 commission. Through its investigations, the commission reported that if the multiple US intelligence agencies had been better integrated, then vital information would have been more readily available, which might have averted the attack (Kean, 2004). Since then, efforts have focused on the “smooth flow of people, ideas, and activities across the boundaries of the intelligence community members” (Director of National Intelligence, 2008, 5).

Inter-agency collaboration may present benefits for information accessibility and for meeting threats; nevertheless, intelligence analysts have expressed reservations about increasing collaboration through AI technologies. In part, this has to do with the nature of classifying data and information as an activity for preserving secrecy on a need-to-know basis. As Galison (2004, 237) writes: “Classification, the anti-epistemology par excellence, is the art of non-transmission.” Problems of collaboration are very much likely to spring from cultural and institutional forces because of the need to preserve secrecy and demonstrate care over information acquired (Vogel et al., 2021, 830). A report commissioned by the US government argued that without adequate cultural and institutional changes to accommodate new AI technology, it will exist either as an underused technology or one that monopolises analysts’ time. In such circumstances, AI may exist as a costly afterthought. The said report provided the example of a federal agency that implemented an AI pilot to generate leads for its investigators to follow up. However, investigators were simultaneously generating their own leads and with limited time for following-up both sets of leads, the investigators “naturally prioritized the leads they had come up with themselves and rarely used the leads generated by AI” (Mitchell et al., 2019, 9).

While this points to the potential practical infeasibility of employing AI, it may be that employing AI is also undesirable. Vogel and colleagues report two sets of reasons from their interviews with intelligence analysts for the undesirability of AI systems. The first is that the reluctance to collaborate can follow the need to retain secrecy or anonymity of a source. Analysts may keep certain information private, refusing to share it openly, to avoid disclosing unintentionally their own identity or that of a source (Vogel et al., 2021). Second, collaboration through AI platforms may mean that tacit knowledge associated with a piece of intelligence and typically verbally communicated by analysts is lost. The problem of contextual understanding is reported by Roff (2020a, 4) who notes that:

“The noisiness of the data and the limitations of the textual extraction and classification leads to significant problems […] In short, the way in which we use AI for events-based coding is also subject to severe limitations because AI cannot understand context from the text it ingests.”

As such, there may be a privileging of types of data-gathering activities as well as privileging of certain types of information to that which systems for augmented intelligence analysis can ingest. As Vogel and colleagues (2021, 835) ask:

“Will these computational systems and technological infrastructures begin to privilege and rely on quantitative, structured data sets for their outputs? What about data from human intelligence, from observation, or other unstructured data not as amenable to codification […] ?”

If there are certain datasets that are not amenable to these systems, this could lead to a narrowing of the kind of data that are prioritised and used by analysts to generate information, which in turn means that valuable information may be overlooked, thereby defeating the reason for employing augmented intelligence analysis. Lastly, intelligence analysts have expressed concerns that greater collaboration may mean that initial meanings or intentions are misconstrued if intelligence reports are shared too early. Analysts often annotate intelligence reports with initial ideas or interpretations. If shared too early without vetting, other analysts may make unwarranted assumptions on the basis of those initial preliminary annotations (Vogel et al., 2021, 833).

More than an ethical problem, this is a cultural one where different cultures, policies, and unspoken rules among intelligence agencies may lead to frictions or mistakes. Addressing this problem requires creating a shared AI culture among collaborating organisations, with similar levels of preparedness, education, protocols, and practices, as well as levels of analyst control over the data shared across an AI system. The recommendation here concerns readiness.

**Make** **organisations** **AI-ready**. Analysts, teams, and organisations should assess their level of readiness to embed AI in their daily tasks. This implies assessing the type of technology on which they can rely, the level of understanding of AI systems in the different teams, and the support structure put in place to optimise the level of readiness, as well as protocols to ensure prompt identification of mistakes, accountability, and redressing mechanisms.

## Conclusion

AI is a tool not fit for every task. Like many other organisations, intelligence agencies should not fall into the techno-solutionist trap, seeing in AI a solution for all the challenges of ensuring the security and defence of democracies. As in many other domains, the use of AI for augmented intelligence should follow a careful strategy and be shaped by governance mechanisms. The strategy should include, for instance, a risk–benefit analysis which examines ethical risks as well as governance mechanisms building on accumulated experience from other domains of AI deployment (e.g. from healthcare to administration of justice) in order to avoid costly mistakes, harms to individual rights, and social injustice.

AI technology has a great potential to aid intelligence agencies and foster more effective and efficient intelligence analysis. This is a potential that must be leveraged. However, for AI to become a structural element of national security processes of democratic societies, it is crucial that this technology is used respecting fundamental values and rights. To this end, organisational awareness of the ethical challenges outlined in this article, the definition and implementation of measures to address these challenges, and overall continuous scrutiny on the ethical implications of using AI for intelligence analysis are necessary requirements. This article is a contribution to meeting these requirements.

## Footnotes

1 This article does not consider other uses of AI such as for “back office” organisational processes or for use in cybersecurity. These uses of AI are distinct from augmented intelligence analysis and require a distinct assessment of the ethical challenges they pose. In a number of areas such as the use of AI for cybersecurity and kinetic defence purposes, there is already a significant body of literature exploring the ethical challenges (see for instance: Khisamova et al., 2019; Blanchard & Taddeo, 2022; Taddeo et al., 2019; Taddeo 2019; Timmers, 2019; Taddeo et al. 2021; Taddeo & Blanchard, 2022a, b; Blanchard, 2023). As noted below, augmented intelligence analysis is a novel area of use of AI which is significantly understudied.

2 Machine learning describes an artificial system able to learn from its environment and improve its performance through feedback mechanisms without the need for additional programming. Deep learning describes the process whereby AI “mimics” the neural networks of the human brain to identify patterns in large datasets. Unless specified, in this article the term “AI” will be used to indicate both these types of AI systems.

3 https://web.archive.org/web/20070613143919/https://www.cia.gov/library/center-for-the-study-of-intelligence/csi-publications/books-and-monographs/analytic-culture-in-the-u-s-intelligence-community/chapter_1.htm

4 https://www.gov.uk/government/organisations/civil-service-intelligence-analysis-profession/about.

5 The definitions of and nature of the relationship between data and information is complex. This simplified definition draws on the account of the intelligence cycle and is used to guide the discussion in this article. However, it should be noted that this is not necessarily a unilinear process. Since AI inputs are “data”, information used for further extraction and processing by an AI system becomes, again, “data” for the AI system at that time.

6 There has been much hype about large language models, but it is important to put recent successes into context. See Bender (2022), Bender and Koller (2020), and Bender et al. (2021) on the limitations of large language models.

7 Cognitive automation can also support surveillance; indeed the developments in AI facial recognition technology may enable “the complete automation of surveillance using CCTV in public places in the near future” (McKendrick, 2019, 2). The anticipated extension in use of facial recognition for surveillance has seen the regulation of these technologies identified as a top priority in the UK National Surveillance Camera Strategy (Biometrics and Surveillance Camera Commissioner, 2017).

8 For a useful outline of “filtering, flagging, and triage” tasks that AI can undertake, see the list provided in a report for Ofcom on AI-augmented content moderation (Ofcom, 2019).

9 The rest of this article does not focus on the ethical problems posed by the deployment of human–machine teaming (HMT) by intelligence agencies, as these problems refer to issues of trust, cognitive autonomy, automation bias, and moral responsibility, which, while related to the topic of this article, are not immediately relevant. The ethical challenges analysed in this article emerge independently from the mode of deployment of AI systems and concern the very nature of AI technology. If anything, the challenges highlighted in this article are only magnified when considering HMT. It is worth stressing that while beyond the scope of this article, future research should be developed on the ethics of HMT and its deployment for national security and defence purposes.

10 A cautionary tale is provided by the 2015 tech start-up PredictifyMe, which entered a partnership with the United Nations (UN) to assess the terrorist risk-preparedness of schools in Pakistan. Gordon Brown, former UK Prime Minister and UN Special Envoy for Global Education, noted that the program would have the capacity to “assess the level of risk preparedness of schools and generate recommendations for school and community safety plans” (Ahluwalia, 2015). PredictifyMe claimed to have developed an AI model able to predict suicide attacks with an accuracy of 72% using 170 data points (Lo, 2015). These results could not be verified, and shortly after entering into partnership with the UN, the firm collapsed (McKendrick, 2019).

11 To put it simply, consider that observing several thousand black ravens (call this evidence x). Then, from x, one could infer the prediction (p) that the next raven observed will be black, or the generalisation (g) that all ravens are black. However, it is quite possible that after observing thousands of black ravens, the next raven observed turns out to be white. So, the inference from x to p, or from x to g, though reasonable, is not true. As inductive inferences have proved to be questionable, it remains to be seen how one can justify use of it.

12 This is sometimes referred to as “blinkered surveillance.”

13 Anderson refers specifically to the Human Rights Act 1998, which gives effect to Article 8 (“Protection of Personal Data”) of the European Charter on Fundamental Rights.

14 This recommendation is consistent with Article 5(1)(b) of the General Data Protection Regulation (GDPR), which states that “personal data shall […] be collected for specified, explicit and legitimate purposes and not further processed in a manner that is incompatible with those purposes; further processing for archiving purposes in the public interest, scientific or historical research purposes or statistical purposes shall, in accordance with Article 89(1), not be considered to be incompatible with the initial purposes (‘purpose limitation’).”

15 More specifically this process focuses on the requirement set for AI systems in the European AI Act (https://artificialintelligenceact.eu), but this is just a specific implementation of the proposed auditing, which is designed to be value-agnostic.

16 https://eur-lex.europa.eu/legal-content/EN/TXT/?uri=CELEX%3A52021PC0206.
